# Comparative evaluation of competency-based and real-patient education in undergraduate clinical clerkships: insights from student feedback and performance

**DOI:** 10.1186/s12909-025-08323-z

**Published:** 2025-11-26

**Authors:** Xi Yang, Qinyi Wu, Haiping Yang, Yu Deng, Feng Chen, Yunfei An, Zhiyong Zhang, Yali Shen, Xuemei Tang

**Affiliations:** 1 Department of Rheumatology & Immunology, Children’s Hospital of Chongqing Medical University, Chongqing, China; 2 Department of Nephrology, Children’s Hospital of Chongqing Medical University, Chongqing, China; 3 Department of Respiratory Medicine, Children’s Hospital of Chongqing Medical University, Chongqing, China; 4 Department of Internal Medicine, Children’s Hospital of Chongqing Medical University, Chongqing, China; 5 Department of Hematology and Oncology, Children’s Hospital of Chongqing Medical University, Chongqing, China

## Abstract

**Background:**

Clinical clerkships are a critical component of medical education, offering students hands-on experience in patient care and an opportunity to apply theoretical knowledge in real-world settings. Traditionally, clinical clerkships have been conducted through real-patient education (RPE), where students interact with actual patients under supervision. However, the introduction of competency-based education (CBE) has shifted the focus towards developing specific competencies through structured and often simulated learning experiences. This study aims to compare these two approaches based on student feedback and comprehensive evaluation to understand their impact on the clinical clerkship experiences of undergraduate medical students.

**Methods:**

This study was conducted at the Children’s Hospital of Chongqing Medical University, Chongqing, China. Students were divided into two groups: one experiencing a CBE integrated with RPE approach and the other a traditional RPE approach. A mixed-methods research approach was employed to leverage the strengths of both quantitative and qualitative dimensions in evaluating the revised pediatric clinical clerkship curriculum. Data were analyzed to compare these two methods. The difference between mean values was calculated using Student’s t-test. A P-value < 0.05 was considered statistically significant.

**Result:**

Results indicate that students in the CBE group generally rated their teaching sessions higher across most metrics compared to those in the RPE group. This suggests that the CBE approach may be more effective in engaging students, enhancing their skills, and increasing overall satisfaction. Moreover, while there was no statistically significant difference in mean exam scores between groups, CBE increased the proportion of students scoring in the 70–89 range on knowledge-based tests and OSCEs.

**Conclusions:**

Our study indicates that the CBE clerkship model was better received and associated with higher student satisfaction. The significant differences in student feedback between the CBE and RPE approaches highlight the need for a review and improvement of teaching methods. An integrated approach, combining the strengths of CBE (structured competency development) and RPE (authentic patient interaction), shows promise in enhancing clinical training. Future studies should employ more robust qualitative methods to further explore the integrative learning experience and long-term outcomes.

**Supplementary Information:**

The online version contains supplementary material available at 10.1186/s12909-025-08323-z.

Clinical clerkships represent a pivotal transition for medical students, bridging theoretical knowledge and clinical practice within supervised healthcare settings. Unlike classroom-based instruction, clerkships uniquely promote the development of communication skills and conceptual understanding through direct patient care. Conventionally, acquiring clinical skills is viewed as an immersive, gradual process, with clerkships serving as the primary arena for practicing and refining these abilities. Through patient encounters in wards and outpatient clinics, students build foundational clinical competency [1, 2]. However, emerging observations suggest that the quantity of clinical exposure during clerkships may not consistently correlate with performance outcomes [3]. This discrepancy invites a re-examination of how clerkships are structured to maximize their educational value.

Competency-based education (CBE) represents an outcomes-focused framework that organizes curriculum, instruction, and assessment around defined, measurable abilities essential for healthcare practice. It aims to align educational outcomes with societal needs for high-quality care by delineating specific competencies that health professionals must master, applicable across medical, nursing, and allied health disciplines [4, 5].

In Chinese medical education, real-patient education (RPE) remains a fundamental and widely used approach in clinical clerkships, prized for its direct interaction with patients and practical exposure. Nevertheless CBE methods are increasingly being implemented, representing a paradigm shift in pedagogical approaches [4]. In 2014, Sun et al. developed the Chinese Doctors’ Common Competency Model, an initiative jointly approved by the National Medical Examination Center and the Ministry of Education [6, 7]. This model has since served as a crucial reference and standard for the training of Chinese medical professionals. Medical education in China is administered through various programs, with Year-4 students starting their clinical clerkships. In Year 5, students attend clinical rotations at teaching hospitals and receive their bachelor’s degree in medicine at the end of their 5th year of study.

Undergraduate medical education is undergoing significant advancements with the introduction of competency-based medical education (CBME). However, studies have not yet fully explored the feasible methods and effects of implementing CBME during the clinical clerkship in Chinese medical education. Equally important is the educational experience, a critical determinant of student engagement, learning efficacy, and development [8]. Student satisfaction is positively correlated with student engagement, and the inclusion of student feedback on assessment practices is essential for informing and refining assessment methodologies within an educational program [3]. Although the literature in higher education underscores the need for an increase in formative assessment to support student learning, there remains a propensity for summative forms of assessment [9, 10].

The pedagogical philosophies underpinning RPE and CBE differ substantially. RPE is rooted in experiential learning within authentic clinical environments, where students develop skills through supervised engagement with real patients. This method prioritizes adaptability to complex, unpredictable clinical presentations and cultivates bedside manner through direct experience [1, 4]. In contrast, CBE employs a structured, outcome-oriented framework. It decomposes clinical competencies into discrete, assessable skills and utilizes standardized simulations to ensure systematic practice, targeted feedback, and mastery of predefined competencies before transitioning to real patients [8–10]. While RPE emphasizes authenticity through direct patient contact, CBE prioritizes standardization, skill repetition, and competency verification in a controlled environment. Our study aims to evaluate the effects of CBE integrated with RPE (hereinafter referred to as CBE) and traditional RPE approach on student feedback and comprehensive evaluation outcomes.

In light of these considerations, this study aims to not only compare the effects of CBE integrated with RPE (hereinafter referred to as CBE) and the traditional RPE approach on student feedback and comprehensive evaluation outcomes, but also to explore how CBE can be effectively incorporated within a real-patient-based curriculum to enhance clinical training. We will place particular emphasis on analyzing qualitative student feedback to better understand the perceived benefits and challenges of the integrated CBE approach. Furthermore, we acknowledge the importance of employing more robust qualitative methods, such as focus groups and interviews, in future research to fully capture the integrative learning experience. These insights will contribute to the ongoing efforts to optimize clinical clerkship design and maximize its educational impact.

## Methods

### Study design and ethics

This study adopted a basic qualitative research design to conduct an in-depth analysis of the experiences of medical students and to examine the processes during their four-month clinical clerkship. A basic qualitative approach is appropriate when addressing practical challenges in educational practice. As it is well-suited to facilitating an in-depth understanding of effective educational processes. By describing medical student’s experiences throughout their clinical clerkship, the study aimed to comprehend and interpret the meanings they attribute to their experiences from their own perspectives.

Pediatrics is recognized as one of the smaller specialties and is therefore allocated a smaller component of the overall curriculum. The clinical medicine program at Chongqing Medical University is divided into general clinical medicine and clinical medicine with a focus on pediatrics. To minimize the impact of these different specializations, we employed both CBE and RPE approach across both groups of students for comparison.

From September 2023 to May 2024, all fourth-year medical students completed their pediatric clinical clerkship, comprising eight sessions covering core pediatric internal systems. The CBE integrated with RPE sessions (total 120 min) were structured sequentially as: (1) 20-minute theoretical review, (2) 30-minute standardized patient activity including history taking, physical examination, and immediate 20-minute group discussion within this time block, (3) 20-minute summary of clinical findings, and (4) 50-minute bedside teaching with real patients. In contrast the traditional RPE sessions (total duration: 120 min) involved: (1) 90-minute immersive bedside patient interaction focused on history taking and physical examination, followed by (2) 30-minute case debriefing. Both approaches maintained equivalent total duration (120 min per session; CBE: 20 + 30 + 20 + 50 = 120 min; RPE: 90 + 30 = 120 min). The number of patients examined per student ranged from 1 to 3 across departments.

Participants consisted of 218 fourth-year medical students from two randomly selected classes (accounting for approximately 50% of the total cohort) undertaking the pediatric clinical clerkship at our institution between September 2023 and May 2024. Prior to the commencement of the clerkship, students were informed of the differences between the CBE and RPE groups and afforded the opportunity to voluntarily elect to participate in the study. All 218 students from the selected classes consented to participate, participation was entirely voluntary, and explicit assurance was provided that refusal would not impact their academic performance. For the administration of the satisfaction questionnaire, teachers allocated 5 min before the end of each class for completion and vacated the classroom to safeguard students’ privacy, with questionnaire participation remaining voluntary. The informed consent form clearly stated that participants retained the right to withdraw their consent retrospectively, and in such cases, all their data would be promptly excluded from analyses and permanently erased from the study database, with no adverse implications for their academic standing. No participants withdrew their consent during or after the study. This study was approved by the Ethics Committee of Children’s Hospital of Chongqing Medical University (registration number: 2024 − 237).

### Evaluation

A mixed-methods research design was adopted to evaluate the acquisition of two core competencies through an assessment framework aligned with the pediatric clerkship curriculum. For Clinical Diagnostic Reasoning Competency, evaluation was conducted via: (1) Pediatric Knowledge-Based Post-Tests featuring case-based questions that required students to analyze pediatric vignettes, identify key symptoms, correlate findings with pathophysiology, and formulate differential diagnoses; and (2) Objective Structured Clinical Examination (OSCE) stations assessing diagnostic logic through systematic interpretation of clinical findings and evidence-based justification of reasoning. For Patient-Physician Communication Competency, assessment comprised: (1) OSCE communication stations utilizing validated checklists to evaluate rapport-building with families, empathetic inquiry, age-appropriate information delivery, and collaborative decision-making; and (2) indirect indicators from the End-of-Rotation Satisfaction Survey capturing student-perceived communication improvement. The OSCE assessment, which incorporates both theoretical knowledge evaluation and practical skills assessment, serves as a standardized and relatively objective measure of clinical skills for fourth-year medical students in China. Similarly, the end-of-term examination also provides a relatively objective evaluation of academic performance. All assessments were administered through the “wenjuanxing” platform (www.wjx.cn), with survey links distributed via WeChat immediately post-clerkship (September 2023-May 2024), ensuring alignment with real-world clinical practice requirements while maintaining voluntary participation through detailed information disclosure.

The CBE curriculum specifically targeted two core competencies with well-defined components. For Clinical Diagnostic Reasoning Competency, the framework included: (1) systematic clinical data collection through history-taking and physical examination; (2) identification of key clinical findings (e.g., distinguishing pediatric symptom patterns); (3) integration of patient data with biomedical knowledge; and (4) formulation of evidence-based differential diagnoses. For Patient-Physician Communication Competency, the components encompassed: (1) establishing rapport with pediatric patients and families; (2) employing empathetic inquiry to understand concerns; (3) delivering medical information using age-appropriate language; and (4) facilitating shared decision-making. These competency components were operationally assessed through the OSCE stations and case-based exam questions to ensure clinical relevance.

The satisfaction survey constitutes a comprehensive instrument designed to assess various dimensions of the teaching process and its impact on students. It consists of several key questions targeting specific dimensions of the teaching experience, with the primary objective of eliciting detailed feedback from students about their perceptions and experiences. Each question offers four response options: A (Strongly Disagree), B (Disagree), C (Agree), and D (Strongly Agree), which are converted into numerical values(A = 1, B = 2, C = 3, D = 4) for quantitative analysis. The survey covers dimensions such as stimulating learning interest, the cultivation of clinical reasoning, improving self-learning ability, enhancing communication and teamwork, the fostering of professional pride, the perception of learning burden, participation enthusiasm, concentration in lessons, mastery of instructional content, and overall satisfaction. This granular feedback serves to assess the instructional effectiveness and identifying areas warranting refinement Clinical medicine students were assessed using a pediatric knowledge-based post-test, whereas pediatric clinical medicine students were evaluated using the OSCE as a tool for assessing clinical performance.

### Statistical analysis

Paired t test was used to compare post-test scores. Scores from the original curriculum were compared with scores from the revised curriculum using Student’s t-test. t-values of less than 0.05 were considered to indicate statistical significance.

## Results

### Number of medical students during clinical clerkship

A total of 218 fourth-year medical students completed the clinical clerkship from September 2023 to May 2024. All students completed the questionnaire. The instructional sessions for students were distributed across the first semester and the second semester of the 2023 academic year. The instructional sessions for general clinical medicine students were scheduled for the first semester, while the instructional sessions for students specializing in pediatric clinical medicine were scheduled for the second semester. A total of 112 general clinical medicine students and 106 pediatric clinical medicine students were included in this study. These students were randomly allocated into two groups: one group received the CBE teaching method, and the other group underwent the traditional RPE teaching method. Among the 112 general clinical medicine students, 67 were assigned to the traditional RPE teaching method, and 45 to the CBE method. Among the 106 pediatric clinical medicine students, 42 were assigned to the traditional RPE teaching method, and 64 to the CBE teaching method.

### Students’ satisfaction towards the clerkship course

Questionnaires assessing students’ attitudes towards the clerkship preparation skills course were distributed to all students who had completed the course after seven course cycles. A total of complete questionnaires were received from students and were included in qualitative analysis. In almost all dimensions of the teaching evaluation scores, the CBE integrated with RPE teaching method performed better than the traditional RPE teaching method. The dimensions with statistically significant differences were: level of participation enthusiasm (*p* = 0.028), concentration (*p* = 0.013), mastery of instructional content (*p* = 0.003), and overall satisfaction (*p* = 0.024) (Table 1).

**Table 1:** Comparison of student perceptions and outcomes between RPE and CBE

Dimension	RPE- Mean	RPE-Std	CBE- Mean	CBE-Std	t-stat	*p*-value
1. The lesson stimulates your learning interest	3.49	0.59	3.61	0.54	−1.68	0.095
2. The lesson expands your clinical thinking	3.53	0.57	3.65	0.53	−1.59	0.112
3. The lesson improves your self-learning ability	3.46	0.59	3.56	0.57	−1.29	0.198
4. The lesson improves your communication and teamwork	3.46	0.6	3.61	0.56	−1.86	0.064
5. The lesson enhances your professional honor as a doctor	3.47	0.6	3.58	0.58	−1.37	0.171
6. The lesson increases your learning burden	2.41	0.96	2.49	0.99	−0.56	0.579
7. Your participation enthusiasm in the lesson	3.29	0.58	3.46	0.52	−2.21	0.028
8. Your concentration in the lesson	3.33	0.59	3.52	0.54	−2.51	0.013
9. Your mastery of the lesson content	3.4	0.56	3.61	0.49	−2.95	0.003
10. Your satisfaction with the lesson	3.48	0.59	3.64	0.48	−2.27	0.024

Abbreviation: *RPE* Real-patient education, *CBE* Competency-based education, *Std* Standard deviation, *t-stat* T-statistic, *p-value* Probability value

### Knowledge-based end-of-rotation exam scores comparison

The mean exam scores for the RPE and CBE groups were 76.63 and 77.62, respectively, with no statistically significant difference (*p* = 0.67). The correlation between satisfaction and exam scores was negligible for both groups, suggesting no significant relationship between student satisfaction and exam performance irrespective of instructional approach. However, a larger proportion of students in the CBE group (86.67%) scored within the 70–89 range compared to the RPE group (79.10%), though this difference did not reach statistical significance (*p* = 0.263) (Table 2).

**Table 2:** Comparison of knowledge-based end-of-rotation exam scores between RPE and CBE

Item	RPE-Group (*n* = 67)	CBE-Group (*n* = 45)	*p*-value
Mean Score	76.63	77.62	0.67
Proportion of scores < 60	4.48% (*n* = 3)	4.44% (*n* = 2)	0.978
Proportion of scores 60–69	11.94% (*n* = 8)	6.67% (*n* = 3)	0.296
Proportion of scores 70–89	79.10% (*n* = 53)	86.67% (*n* = 39)	0.263
Proportion of scores > 90	4.48% (*n* = 3)	2.22% (*n* = 1)	0.675

Abbreviation: *RPE* Real-patient education, *CBE* Competency-based education, *p-value* Probability value

### OSCE exam scores comparison

The OSCE exam scores between RPE and CBE groups showed comparable performance, with mean scores of 81.22 (RPE) and 82.67 (CBE) (*p* = 0.196). The correlation between satisfaction and OSCE scores remained negligible in both groups, indicating no significant relationship between satisfaction and exam performance irrespective of the instructional approach. Notably, while not statistically significant (*p* = 0.365), a higher proportion of CBE students (89.06%) scored within the 70–89 range compared to the RPE group (83.33%) (Table 3).

**Table 3:** Comparison of objective structured clinical examination (OSCE) scores between RPE and CBE

Item	RPE-Group (*n* = 42)	CBE-Group (*n* = 64)	*p*-value
Mean Score	81.22	82.67	0.196
Proportion of scores < 60	0 (*n* = 0)	0 (*n* = 0)	1.000
Proportion of scores 60–69	14.29% (*n* = 6)	7.81% (*n* = 5)	0.286
Proportion of scores 70–89	83.33% (*n* = 35)	89.06% (*n* = 57)	0.365
Proportion of scores > 90	2.38% (*n* = 1)	3.13% (*n* = 2)	1.000

Abbreviation: *RPE* Real-patient education, *CBE* Competency-based education, *p-value* Probability value

## Discussion

Clinical clerkships form a cornerstone of medical education, providing indispensable hands-on experience in patient care and the application of theoretical knowledge. The traditional RPE approach, centered on supervised interactions with actual patients, offers exposure to the complexities and uncertainties inherent in real-world medicine, fostering the development of bedside manner and adaptive clinical decision-making [8]. In contrast, CBE prioritizes the attainment of specific, predefined competencies via structured, often simulated learning sequences. This model emphasizes skill mastery within a more controlled and standardized environment, frequently incorporating simulated patients and scenarios to allow for deliberate practice and immediate feedback [4, 11]. A central challenge in clinical education is how to effectively merge the authentic immersion of RPE with the systematic skill-building of CBE to optimize training outcomes, a question that motivates the present investigation.

Our study indicated that students who received CBE integrated with RPE generally assigned higher ratings to their instructional sessions across most evaluation metrics compared to those who experienced traditional RPE. This suggests that the CBE approach may be more effective in engaging students, enhancing their skills, and increasing overall satisfaction. One of the notable findings was that CBE increased the proportion of students scoring in the 70–89 range on tests. This indicates that the structured and goal-oriented nature of CBE might better prepare students for evaluations compared to the more variable real-patient interactions in RPE [3]. These findings underscore the value of integrating CBE within a real-patient-based curriculum, combining structured skill development with authentic clinical exposure to maximize educational impact.

CBE’s approach provides several advantages. First, it facilitates the identification and targeted remediation of specific weaknesses, ensuring that all students attain a baseline level of proficiency. The practice and immediate feedback inherent in CBE enhance learning and retention of skills [2]. Specifically, in Clinical Diagnostic Reasoning Competency, students systematically collect patient information (history-taking, physical examination), analyze data, and deduce diagnoses. This involves identifying key clinical findings, integrating data with medical knowledge, and formulating evidence-based differential diagnoses. In the CBE group, students practiced through complex real or simulated case analyses, with structured discussions and feedback to refine reasoning—assessed via case-based final exam questions and OSCE stations evaluating diagnostic logic.

Additionally, CBE ensures exposure to a wide range of clinical scenarios through standardized patients, addressing variability limitations in RPE [11]. A key component is Patient-Physician Communication Competency, which focuses on establishing trust, eliciting concerns, and collaborating on decisions through empathetic inquiry and age-appropriate communication. CBE students honed these skills via role-plays and supervised real-patient interactions, learning active listening and clear explanation techniques. Competency was assessed using OSCE communication stations (validated checklists) and final exam evaluations, aligning training with real clinical demands.

The increased student satisfaction and engagement with CBE indicates that this approach may be more congruent with the learning preferences of contemporary medical students, who often appreciate clear goals, structured learning environments, and the opportunity to receive and act on feedback promptly. These aspects of CBE can create a more supportive and effective learning environment, which is crucial for developing competent and confident medical professionals [10].

While RPE offers the invaluable experience of real-patient interactions, it also comes with challenges. The variability and unpredictability of clinical cases can pose challenges in ensuring that all students obtain a comprehensive and well-rounded clinical education. Some students may not encounter certain conditions or procedures, leading to gaps in their training. Moreover, the reliance on real patients can limit the volume of hands-on experience a student can gain, particularly in high-risk situations where patient safety is paramount [9]. Despite the clear advantages of CBE, it is essential to recognize that real-patient interactions are irreplaceable in certain aspects of medical training. Therefore, an integrated approach that combines the strengths of both CBE and RPE could offer the most comprehensive educational experience. By incorporating simulation-based modules and structured competency assessments within the traditional RPE framework, medical education programs can ensure that students benefit from the best of both worlds [10].

However, to fully elucidate the mechanisms behind the effectiveness of integrated CBE, future studies should employ more robust qualitative methods—such as focus groups and in-depth interviews—to capture the nuances of student experiences and perceptions. Future research should examine the long-term impacts of CBE and RPE on clinical competence and patient outcomes. Studies could also explore the optimal balance between simulated and real-patient experiences in medical education. Additionally, investigating the scalability of CBE in diverse educational settings and its effectiveness across different medical specialties could provide valuable insights for curriculum development.

### Strengths and limitations

This study offers several strengths, including its targeted focus on a critical component of medical education-pediatric clinical clerkships-by systematically comparing CBE integrated with RPE and traditional RPE, which addresses a practical need for optimizing clinical training methods. The mixed-methods approach, combining quantitative data (satisfaction surveys, knowledge-based tests, OSCE scores) with statistical analyses (Student’s t-test), and attention to ethical rigor (voluntary participation, consent processes, privacy protection) enhance the validity of findings. Additionally, detailed measurement of satisfaction across multiple dimensions provides nuanced insights into how pedagogical approaches influence student experiences.

However, limitations exist: the sample, consisting of 218 students from 2 randomly selected classes at a single institution (≈ 50% of the grade), limits generalization to other educational contexts. The 4-month follow-up period and immediate post-clerkship assessments lack long-term data on the persistence of observed differences. Discrepancies in real-patient contact time (50 min for CBE vs. 90 min for RPE) may confound comparisons. Many outcomes (e.g., exam score differences) lacked statistical significance, potentially due to limited sample size. Over-reliance on short-term, subjective surveys and narrow assessments (tests, OSCE) may not capture deeper competencies, and the mixed-methods design prioritized quantitative data, with minimal qualitative exploration of students’ lived experiences, restricting understanding of why CBE performed better in specific dimensions.

## Conclusion

In summary, our study demonstrates that the demand for CBE clerkship is substantial. The significant differences in student feedback between the CBE and RPE approaches highlight the need for a review and improvement of teaching methods. By incorporating effective practices from the more successful CBE approach and targeting areas requiring improvement, we can enhance the quality of the clinical clerkship experience, ultimately yielding leading to better-prepared medical professionals and greater overall satisfaction.

## Supplementary Information

Supplementary Material 1.

Supplementary Material 2.

## Data Availability

The study materials and the detail of all analyses are available from the corresponding author upon reasonable request.

## Abbreviations

RPE: Real-Patient Education
CBE: Competency-Based Education
CBME: Competency-Based Medical Education
OSCE: Objective Structured Clinical Examination

## References

[1] Dornan T, Conn R, Monaghan H, Kearney G, Gillespie H, Bennett D. Experience based learning (ExBL): clinical teaching for the twenty-first century. Med Teach. 2019;41(10):1098–105. 31382787 10.1080/0142159X.2019.1630730

[2] Yardley S, Teunissen PW, Dornan T. Experiential learning: transforming theory into practice. Med Teach. 2012;34(2):161–4. 22288996 10.3109/0142159X.2012.643264

[3] Ten Cate O, Scheele F. Competency-Based postgraduate training: can we bridge the gap between theory and clinical practice? Acad Med. 2018;92(6):676–83. 10.1097/ACM.0b013e31805559c7 17525536

[4] Frank JR, Snell LS, Ten Cate O. Competency-Based Medical Education: theory to practice. Med Teach. 2020;42(1):12–8. 10.3109/0142159X.2010.501190 20662574

[5] Holmboe ES, Yamazaki K, Edgar L, Conforti L, Holmboe L. Reflections on the first 2 years of milestone implementation. J Grad Med Educ. 2019;11(4):622–4. 10.4300/JGME-07-03-43 PMC4597976 26457171

[6] Sun H, Zhang Y, Li Y. The development of the Chinese doctors’ common competency model. Chin Med J. 2014;127(23):4095–9.

[7] National Medical Examination Center. Zhongguo linchuang yisheng gangwei shengrenli moxing goujian yu yingyong [Construction and application of the competency model for Chinese clinical physicians]. People’s Medical Publishing House; 2015.

[8] Nicol DJ, Macfarlane-Dick D. Formative assessment and self-regulated learning: a model and seven principles of good feedback practice. Stud High Educ. 2006;31(2):199–218.

[9] Gikandi JW, Morrow D, Davis NE. Online formative assessment in higher education: a review of the literature. Comput Educ. 2011;57(4):2333–51.

[10] Lockyer J, Carraccio C, Chan MK, Hart D, Smee S, Holmboe ES. Core principles of assessment in competency-based medical education. Med Teach. 2017;39(6):609–16. 28598746 10.1080/0142159X.2017.1315082

[11] Lurie SJ, Mooney CJ, Lyness JM. Measurement of the general competencies of the Accreditation Council for Graduate Medical Education: a systematic review. Acad Med. 2017;84(3):301–9. 10.1097/ACM.0b013e3181971f08 19240434

